# On the Function of the Spleen and Other Lymphatic Glands, as Secretors of the Blood

**Published:** 1853-01

**Authors:** J. H. Bennett


From the London Monthly Jour. of Med. Science.

*On the Function of the Spleen and other Lymphatic Glands, as Secretors of the Blood, by* J. H. Bennett, M. D.

1. *Relations between the Colorless and Colored Corpuscles.* Dr. Bennet, believes, with Mr. Wharton Jones, that the colored corpuscle is merely the liberated nucleus of the colorless cell. The transformation takes place in the following manner:—The colorless cell may frequently be seen, by the aid of acetic acid, to have a single round nucleus; but more commonly the nucleus is divided, each half having a distinct depression, with a shadowed spot on the centre. Occasionally, before division takes place, the nucleus becomes oval, elongated, and sometimes bent, or of a horse-shoe form It may be divided into three or four granules. These stages are figured by Dr. Bennet; they were discovered by him in his interesting observations on leucocythemia, and in experiments on mammals, birds, reptiles and fishes.

He does not believe, with Mr. Wharton Jones, that all the nuclei forming the colored corpuscles, in mammals, should necessarily be provided with a cell-wall. Many, however, do proceed beyond this point, and may be seen to have cell-walls; the nuclei, in such cases, increase endogenously, by fissiparous division, and, on the solution of the cell-wall, become colored blood-discs. In fishes, reptiles and birds, the colored blood-corpuscles are nucleated cells, originating in the blood-glands.

2. *Origin of the Blood-Corpuscles.* This (as was enunciated many years ago by Hewson) is to be looked for in the lymphatic glandular system, under which head are included the spleen, thymus, thyroid, supra-renal, pituitary, pineal and lymphatic glands. Nuclei and nucleated cells are found in these bodies, and Dr. Bennett's observations on leucocythemia have shown that an increase of colorless cells in the blood is connected with enlargement of the spleen and other glandular organs. The blood of the splenic and portal veins is always richer in colorless corpuscles than that of the systematic circulation; and in young animals, in which the thyroid, thymus and supra-renal glands are most fully developed, the blood contains most colorless corpuscles. Moreover, in a case of enlargement of the thyroid body, this organ contained cells and nuclei of much smaller size than usual, and corresponding cells and nuclei were found in the blood. In another case, the colorless corpuscles in the blood were of two distinct sizes, corresponding with a similar appearance in the corpuscles of the lymphatic glands. It is difficult to determine how the corpuscles find their way from the lymphatic glands into the blood; but Dr. Bennett suspects that there must be a direct venous communication. He believes that, if he has established that the corpuscular elements in the so-called blood-glands are transformed into those of the blood, it will follow that the lymphatic glands secrete the blood-corpuscles the same as the testes secrete the spermatozoa, the mammæ the globules of the milk, or the salivary and gastric glands the cells of the saliva and gastric juice.

The most probable and consistent mode of origin of the corpuscles is in an organic fluid, by the production of molecules, the successive development and aggregation of which constitute the higher formations. Multitudes of free nuclei join the blood, and are at once converted into colored blood-discs; and their cells circulate for a time, when their walls are dissolved and their nuclei become colored. The number of colored corpuscles in the blood increases in proportion to the development of the lymphatic glandular system in the animal kingdom, and Mr. Drummond and Dr. Bennett have observed that the nuclei in the spleen, varying in size in different animals, correspond with the nuclei of the blood-corpuscles.

3. *Ultimate Destination of the Blood-Corpuscles.* Dr. Bennett believes that the blood-corpuscles are dissolved, and, with the effete matter absorbed from the tissues of the lymphatics, constitute blood-fibrin. Zimmerman believed that fibrin resulted from the metamorphosis of the textures. The arguments which support this view appear to Dr. Bennett to be unanswerable. There is no fibrin in the chyme, very little in the chyle, less in carnivora than in herbivora. There is no fibrin in the egg, nor in the blood of the fœtus, and very little in the new-born infant. On the other hand, all those circumstances which cause exhaustion of the textures, or increase the amount of absorption, augment the quality of fibrin; as after inflammatory or other exudations, starvation, violent fatigue, pregnancy, and frequent bleeding and hæmorrhage. The amount of fibrin in the blood seems out of proportion to what would be required for textural nutrition. Increase of fibrin is also accompanied with diminution of the red corpuscles: hence it appears probable that fibrin results from a solution of the blood-corpuscles, conjoined with the effete matter derived from the secondary digestion of the tissues, which is not converted into albumen.

